# Next-of-kin involvement in improving hospital cancer care quality and safety – a qualitative cross-case study as basis for theory development

**DOI:** 10.1186/s12913-018-3141-7

**Published:** 2018-05-03

**Authors:** Inger Johanne Bergerød, Bjørnar Gilje, Geir S. Braut, Siri Wiig

**Affiliations:** 1 Stavanger University Hospital, Gerd-Ragna Bloch Thorsens gate 8, 4011 Stavanger, Norway; 2 SHARE - Center for Resilience in Healthcare, Faculty of Health Sciences, University of Stavanger, Stavanger, Norway

**Keywords:** Case study, Next-of-kin, Family, Caregiver, Quality, Safety, Quality improvement, Cancer, Hospitals, Cross case analysis, Theory development

## Abstract

**Background:**

Next-of-kin are an extension of healthcare professionals in all stages of cancer care. They offer care activities such as interpretations of symptoms, and reporting of negative or adverse effects of treatment, without any professional knowledge or skills. Their participation is often expected from healthcare professionals, managers, or the patient. However, there is limited knowledge of next-of-kin’s role in and contribution to quality and safety improvement in hospital cancer care. The aim of this study was to explore how managers and healthcare professionals understand the role of next-of-kin in cancer care, and what methods they use for next-of-kin involvement.

**Methods:**

The study design was a comparative multiple embedded case study of cancer departments in two Norwegian university hospitals. Data collection methods consist of qualitative interviews with managers (13) and healthcare professionals (19) collected in 2016, and document analysis of policy documents and regulation. The interviews were analyzed according to a directed content analysis approach guided by the theoretical framework ‘Organizing for Quality’.

**Results:**

Both hospitals have a strategy to involve next-of-kin in treatment and care but have no formal way of doing so. Managers and healthcare professionals in the two hospitals illuminated nine areas where next-of-kin are important stakeholders in improving quality and safety. These nine areas (e.g. nutrition, observations, transitions, pain treatment, information, palliative and terminal care) are common across the two hospitals. Key challenges in the next-of-kin involvement pertain to insufficient physical working conditions and room facilities, and lack of continuity of experienced nurses and consultants.

**Conclusion:**

Hospital employees and managers regard next-of-kin as a safety net or a buffer that cannot be replaced by other stakeholders. This study shows a close collaboration between patient, next-of-kin and healthcare professionals in cancer care, but more effort should be invested in more systematic approaches for next-of-kin involvement in quality and safety improvement such as a guide for managers and healthcare professionals on methods and areas of involvement.

## Background

Next-of-kin and especially family caregivers are an extension to the healthcare professionals and are often involved in all stages of the cancer care trajectory [1, 2]. Next-of-kin often provide care such as interpretations of symptoms, and reporting of negative or adverse effects of treatment, without any professional knowledge or skills. This participation is often expected from healthcare professionals, managers, the patient or significant others [3, 4]. A systematic literature review has identified more than 200 problems related to caring for cancer patients [5]. The Institute of Medicine [6] has highlighted next-of-kin as an important safety dimension in patient-centered care. A study published in 2017 concluded that cancer patients experience adverse events more often than other hospitalized patients [7].

There are limited descriptions of the methods and challenges in involving next-of-kin in improving the quality and safety of hospital cancer care [4]. A few studies on the role of next-of-kin in cancer care have demonstrated that some of the reasons for close interaction between next-of-kin and patients are related to quality and patient safety concerns. Sapountzi-Krepia and colleagues [8] revealed in their study that next-of-kin in a cancer hospital remain at the bedside because of the severity of the patient’s condition; to provide psychological support; as a family tradition; because they did not believe that the patient was safe in the hospital; and because of shortage of healthcare professionals.

In Norway the government has launched a change in the next-of-kin policy including stronger involvement of next-of-kin in healthcare [9, 10]. The aim is to pay more attention to the interaction between the next-of-kin and the healthcare services to improve quality and safety of healthcare. A major concern is the lack of voluntary caregivers and recruitment of healthcare workers in the future. It is therefore crucial to acknowledge the next-of-kin expertise and explore their role [11].

Studies in other areas than cancer care such as transitional care [12, 13], elderly care [14, 15] and pediatrics [16, 17] have explored the influence of next-of-kin on quality and safety in healthcare. Jeffs and colleges results pointed out that the caregivers often become substitutes for adequate staffing and that future research should provide insight in how to best engage caregivers actively in care transitions. Storm and colleagues documented that quality was impaired by the lack of systematic information exchange between healthcare professionals and next-of-kin, and by the limited involvement and preparation of patients and next-of-kin for transitions across care levels within elderly care. Next-of-kin were bridging between the patient and healthcare professionals, they were patient advocates and supporters, and contributed to information brokering between the healthcare providers and the patient. Moreover, Rustad (2017) highlighted that next-of-kin provided important information about the patient’s health, and supported the patient’s self-care in the field of transitional care of the elderly [14]. Other previous studies have shown how family caregivers provide valuable information that improved safety for pediatric inpatients [16], and Davis and colleagues [18] highlighted predictors of healthcare professionals’ attitudes towards next-of-kin involvement in quality improvement. In particular, a discouraging response from healthcare professionals decreased the support for next-of-kin involvement and had strong perceived negative effects on next-of-kin relationship with healthcare professionals [18]. Furthermore, some studies show that next-of-kin takes on several tasks they are unprepared to handle, often resulting in higher caregiver burden [19, 20].

Previous research indicates that there is limited knowledge about the healthcare professionals` and managers` perspective on involvement and the role of next-of-kin in cancer care. Moreover, there is a need to explore the division of work between healthcare services and next-of-kin to reduce burden, and to ensure a sustainable involvement in quality and safety improvement in hospital cancer care [1, 2].

### Aim and research question

The aim of this study was to explore the influence of next-of-kin involvement on quality and safety improvement within cancer care in hospitals. This study also explored how managers and healthcare professionals understand the role of next-of-kin in cancer care, and what methods they use for next-of-kin involvement. The following research questions guided the study: How are next-of-kin involved in hospital cancer care? How do managers and healthcare professionals perceive challenges in next-of-kin involvement in cancer care?

By studying national policy documents and qualitative interviews with managers and healthcare professionals, this study contributes to a better understanding of the diversity and complexity of next-of-kin involvement in cancer care, and deepens the understanding of how the relationship between the patient, next-of-kin and healthcare services can improve the service quality and safety in this field.

## Methods

### Design and setting

This article is the first in a larger mixed-method convergent design study [21]. The purpose of a convergent design is to collect and analyze quantitative and qualitative data separately and merge the two in order to compare the results [21]. The study design in this article is a comparative multiple embedded case study of cancer departments in two Norwegian hospitals. A case is defined as a hospital and the belonging cancer departments. The case is embedded, meaning that it includes several units of analysis (macro, meso, micro level) [22]. It includes managers at the meso level and healthcare personnel at the micro level. In addition, we use national policy documents and regulations to illustrate the macro level context. A case study research strategy is chosen because the phenomenon of next-of-kin involvement in hospitals` cancer care improvement is a complex process involving activities of daily operations of a hospital and cannot be explored in isolation from each other. Through the empirical material the purpose is to gain insight into the relation between next-of-kin interaction and its influence on quality and safety improvement. The two hospitals have been explored separately at the meso level (department managers) and at the micro level (healthcare professionals), within the respective cancer departments. The comparative design seeks the meaning of the similarities and differences in involvement and the challenges between the hospitals.

### The case hospitals

Two cancer care departments at two university hospitals within one regional health authority (RHA) in Norway, constitute the studied cases. The two hospitals differ in size, employees and budget (Table 1), but are subject to the same national and regional policy documents.

**Table 1:** Contextual Description of the Two Cases

Context	Hospital A	Hospital B
Localization	Large city in Norway	Large city in Norway
Case hospital	University hospital Local hospital for 330.000 inhabitants Second largest regional cancer department	University hospital Local hospital for 420.000 inhabitants Largest regional cancer department
Employees	7500	12,000
Budget	6,8 billion NOK	10,8 billion NOK

Hospital A is the second-largest university hospital in the RHA. Its cancer department consists of two cancer care wards (40 beds), two outpatient clinics, and one radiation therapy unit. The outpatient clinics offer approximately 750 chemotherapy treatments per month. Hospital B is the largest university hospital in the RHA. This cancer department is the main regional cancer clinic. The cancer department at Hospital B consists of two inpatient wards, one outpatient clinic and one radiotherapy unit. Both departments have seen an increased amount of treatment and patient throughput in the last few years, and are consistently working to meet this challenge.

### Data collection

The study applies several data sources. National policy document such as regulations, and reports to the parliament were collected and analyzed to explore the macro level context with focus on demands and expectations for next-of-kin involvement in general [10, 11] and, in cancer care [23] and patient safety [24]. At the meso level we conducted qualitative interviews with managers and, collected and analyzed hospital strategy documents. At the micro level we conducted qualitative semi-structured interviews with healthcare professionals in the two hospitals. Thirty-two semi-structured interviews were conducted over a four- month period (December 2015 to March 2016). All informants were recruited by their nearest manager using snowball sampling to identify additional informants. All managers and healthcare professionals belonging to the departments could be included as informants. Only one of the approached informants declined the invitation. Table 2 shows the total number of informants in Hospitals A and B.

**Table 2:** Total Number of Informants in the Two Hospitals

Hospital A		Hospital B	
Meso level (managers)		Meso level (managers)	
Consultant	1	Consultant	2
Nurse	2	Nurse	–
Oncology nurse	3	Oncology nurse	4
Quality manager	1	Quality manager	–
Micro level (healthcare professionals)		Micro level (healthcare professionals	
Consultant	2	Consultant	2
Nurse	4	Nurse	2
Oncology nurse	3	Oncology nurse	6
Total	16	Total	16

All informants received information explaining the purpose of the study, methods, limitations, and what role they were expected to play and the possible outcome of the research. To ensure that the information was understood, we appointed a local coordinator in both hospitals to give information, and respond to any questions. All informants signed informed consent and we ensured to pinpoint in the startup session in each interview that it was voluntary to participate in the study, to avoid any ethical dilemma for the informants given that managers were involved in the recruitment.

Interview guides were developed based on the theoretical framework ‘Organizing for Quality’ [25]. Several theoretical models can be applied to guide quality improvement and patient safety work in hospitals [26]. Most of them mention organizational structure, leadership, culture, politics, work conditions, and learning to understand how hospitals organize for quality and patient safety [27]. The conceptualization of quality and safety of cancer care in this study supports Bate et al. (2008) including patient safety, clinical effectiveness and patient centeredness [25, 26]. The interview guides also included questions about conceptualization of quality and safety. Bate and colleagues focus on six challenges that need to be addressed in quality and safety improvement work. These are listed as topics in the interview guides. The six challenges are:

1. Structural – organizing, planning and coordinating quality efforts;
2. Political – addressing and dealing with the politics of change surrounding any quality improvement effort;
3. Cultural – giving ‘quality’ a shared, collective meaning, value and significance within the organization;
4. Educational – creating a learning process that supports improvement;
5. Emotional – engaging and mobilizing people by linking quality improvement efforts to inner sentiments and deeper commitments and beliefs;
6. Physical and technological – the designing of physical systems and technological infrastructure that supports and sustains quality efforts ([25], p.169).

The reason for applying the Organizing for Quality framework is based on a need for a system wide and multilevel perspective taking into account inner and outer context of the organization to help understand quality and safety processes [26]. The Organizing for Quality framework has been developed based on international studies of leading hospitals with success in quality improvement. Moreover, the framework has been applied in studies of Norwegian hospitals [28–30], in international studies [26, 31, 32] and as a foundation for a guide for hospital managers’ work to improve quality and safety [31]. A systematic review of quality improvement models in healthcare from 2009 highlights that there is no single framework that stands out above the others. The key to success depends on the understanding of the interaction between the local context and the approach that is applied [33].

### Analysis

**Fig. 1:**
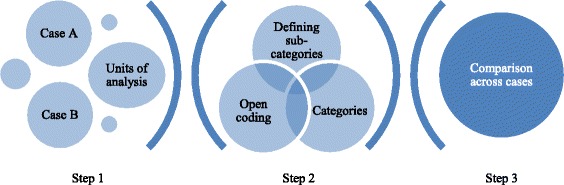
Inductive data analysis procedure in three steps inspired by [43]

Qualitative content analysis is one of many methods used for analyzing qualitative data. We have used directed content analysis inspired by Hsieh and Shannon (2005) [34] guided by the Organizing for Quality framework [25]. The goal was to determine the relationship between the six challenges described by Bate and colleagues and our research questions and to extend the empirical testing of the model. All members of the research team participated in the analysis using group consensus to strengthen validity of our findings [32]. IJB further developed the analysis with several iterations with all authors. Analysis began with the inductive approach (Fig. 1) to capture the essence of next-of-kin involvement in cancer care. Each member of the group did a three-step interpretive characterization of the two cases (Fig.1). Step 1: selecting units and levels of analysis; Step 2: open coding from plain text, defining categories and sub categories; Step 3: comparison of findings across cases and levels.

Findings of the inductive analysis are not the main focus of this article, but were an important part of the analytical validation of the results. Relevant policy documents were approached through close reading, searching for expectations related to involvement of next-of-kin.

**Fig. 2:**
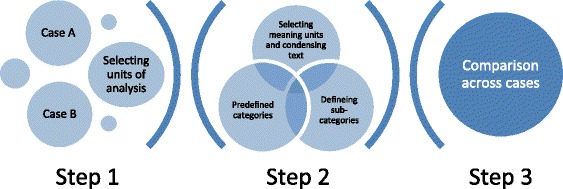
Deductive data analysis procedure in three steps inspired by [43]

In addition, the interview data was categorized according to the six challenges. In the deductive part of the analysis (Fig. 2) the research team met three times to discuss findings using the predetermined codes in the Organizing for Quality framework. The analysis followed a three-step model (Fig.2) within the six challenges in each case hospital, across organizational levels within each case, and across the two case hospitals:

Step 1: Selecting units and levels of analysis;

Step 2: Organizing data with predefined categories; discovery of meaningful units in plain text; condensing short summery of data from the informants; describing subcategories;

Step 3: Description of findings within the six challenges, across levels and cases.

The deductive analysis using the Organizing for Quality Model corresponded well with our findings. There is always a risk that a predefined framework may bias the analysis. Our combination of an inductive and deductive approach contributed to prevent that the Organizing for Quality framework forced the analysis procedure in the deductive part. Using an analytical approach involving only a prior framework could imply omitting key findings emerging from results if they are not applicable in a predefined framework. In this study, we also argue that the six challenges in the Organizing for Quality framework are broad in scope and imply that the results contribute to give content to the challenges from a cancer context perspective. Relating the findings to the governing documents for specialized healthcare in Norway expands the perspective even more, as policy expressions deals with identical issues. The findings thus may be relevant for analysis and discussion irrespective of our chosen framework.

## Results

The results are presented according to the six challenges in the result section. First, we start by presenting the national policy context. Then we present each challenge according to the Organizing for Quality framework with description of sub-categories. In the end of the result section we present a model for important areas of next-of-kin involvement that emerged by seeing the data material as a whole.

### Context – National policy

The last 50 years the Norwegian healthcare sector has been characterized by an increasing public engagement in the provision of health care with generous benefits for the individual inhabitant when in need of healthcare [35]. The formal expectations for participation, economically and practically, by the family or members of the private network of a patient, are low.

There is no statutory obligation for next-of-kin to provide care, but according to the national strategy documents there is an expectation that it should be done either by love, citizenship or by duty. In the National Cancer Strategy in Norway [23], next-of-kin involvement is one of five objectives that are reported to improve safety. In 2013 the Norwegian board of Health Supervision conducted a risk analysis of cancer care [36]. One of top 16 patient safety hazards in this analysis is lack of involvement of patients and their next-of-kin. Results from the analysis of national policy documents show that there is a limited focus on the healthcare professional and managerial views on next-of-kin involvement, not only to support next-of-kin, but also to explore in what ways next-of-kin contributes to quality and safety in hospital cancer care.

### Structural challenges

#### Lack of systematic approaches for next-of-kin involvement

In the two case hospitals, managers and healthcare professionals recognized next-of-kin as important supports in the cancer care trajectory. Interaction and next-of-kin collaboration was on the daily agenda. There was a holistic and respectful attitude to next-of-kin who are considered no less important than the patient, and natural collaboration partners. This was manifested in a written strategy in hospital A. In addition, hospital A adopted a value-based leadership which was known as ‘Respect for all’. The latter seems successful in this hospital, and gave all employees a common vision, values and goal for treatment and care. Both hospitals’ cancer departments valued next-of-kin involvement in different ways, but there was no systematic approach, or plan to operationalize a strategy for next-of-kin involvement. Managers and healthcare professionals at both sites insisted that a more structured way of guidance, such as a checklist in next-of-kin involvement, and a way of collecting information on next-of-kin experiences could improve the role of next-of-kin in improving care quality and safety. Managers and healthcare professionals at both sites were evocative of the national policy and the growing awareness of more next-of-kin involvement in care. Even if both hospitals had a strategy to enlist next-of-kin in treatment and care, there are few formal ways of doing so. Both managers and healthcare professionals claimed that they depended on practical assistance and supervision of the patient from next-of-kin to improve quality and safety of cancer care.

Some methods and tools were identified in relation to collecting next-of-kin experiences. Among these were a questionnaire to next-of-kin, user surveys, and documentation of conversations with children. Both sites encouraged patients in summon letters to bring next-of-kin, and both managers and healthcare professionals often offered next-of-kin meetings or information phone calls. In addition, both hospitals offered unlimited phone hours, and both patient and next-of-kin could call in if they encountered difficulties after discharge.

We are offering next-of-kin conversation or family meetings all the time if the patient wants to bring their next-of-kin. (Consultant)

Most methods focused on how to inform next-of-kin, but the methods did not have systematic means of guidance or educating next-of-kin in knowledge, attitude and practices relating to quality and safety.

#### Next-of-kin as quality and safety resources

The managers and healthcare professionals in both hospitals insisted that next-of-kin constitute important safety resources during treatment:

Next-of-kin are very important during the course of treatment. For example, how safe it is for the patient to go home in neutropenic phase depends on whether they live alone or if they have careers who can act, help and support. Next-of-kin are a very important piece in addition to all emergency personnel in the municipalities, such as nurses, consultants, mobile palliative care team, nursing homes, or homecare services. (Consultant)

Results showed that healthcare professionals depended on collaboration with next-of-kin during patient discharge. Next-of-kin and especially family caregivers often had an important role that could not be replaced by professional healthcare workers. In addition, next-of-kin were considered an invaluable safety resource when the patient was frightened, anxious, restless, or if the facility was understaffed. Healthcare professionals also claimed that without next-of-kin present to help with a patient’s feeding, observation or support it would be difficult to take proper care of them:

If it wasn’t for next-of-kin, the schedule would be disrupted. That could affect other patients with delayed medical care, food, and personal care. (Cancer nurse)

#### Lack of continuity reduces next-of-kin involvement

In both hospitals, managers and healthcare professionals argued that lack of experienced nurses and consultants were obstacles to next-of-kin involvement. This was described as the largest structural challenge to patient safety. In addition, it was more difficult to involve next-of-kin if the consultants did not know them or the patient. Next-of-kin have also complained about lack of continuity of care at both sites:

We have received letters from both patients and next-of-kin who argue that it is tiring to deal with new faces every time they come to the clinic. They come every 14 days, and haven’t seen the same consultant in the last 16 weeks. It is pretty bad! (Registrar)

### Political challenge

#### Lack of interdisciplinary collaboration hampers next-of-kin involvement

Interdisciplinary collaboration is a success factor for next-of-kin involvement in both hospitals. However, the results showed problem with interdisciplinary collaboration, especially in Hospital A. Because of a lack of consultants, nurses felt obligated to take responsibility for tasks such as giving information about treatment options. The nurses expressed frustration over the lack of interdisciplinary arenas, while the consultants seldom acknowledge their own role in interdisciplinary meeting arenas:

We had a patient who died in a lot of pain and we felt that we had failed in some ways, or that we were unable to help the way we wanted to, even if we spent a lot of time with the consultants in the palliative team. Then the nurses conducted a debrief and we were invited to sit in to talk about it. We don’t have time to do so in the consultant group, I think was the idea then… (Registrar)

In Hospital B, registrars are rotated according to the day’s resource needs. As a result, registrars often discharged patients they had never met before. In addition, registrars described that it was common to discharge up to ten patients a day, in addition to taking rounds. This workload made them unable to take the opportunity to learn from role models by joining consultants as they were giving information about treatment or prognosis to patients and next-of-kin.

Nurses and consultants in both sites were led by a manager that had no authority beyond their professional group. Result showed interdisciplinary differences in the conceptualization of quality and safety in cancer care services between the professional groups on what the patient or next-of-kin should know before initiating treatment.

#### The difficult duty of confidentiality

Also important is maintaining confidentiality. Managers and healthcare professionals at both sites explained that next-of-kin should only be informed if the patient consents. At the same time, healthcare professionals claimed that next-of-kin in cancer care often have their own need for information, support, and guidance. Healthcare professionals spend a lot of time responding to requests from the next-of-kin. This activity was seldom documented, but it was important in terms of the close relationship between stakeholders following a cancer diagnosis. This was also an essential follow-up in terms of tasks delegated to the patient and next-of-kin when the patient is between treatments when confidential patient information was required.

### Cultural challenge

#### Next-of-kin as an equal partner and a practical resource

In both hospitals, managers focused on building a collective culture with a holistic approach emphasizing that the whole family is affected when a person is diagnosed with cancer. The managers argued that they had to keep working on this, especially when new employees are hired, since the culture – for better and worse – is learned quickly:

That has something to do with safety. That you dare to stand in to do difficult and tough tasks. To answer questions and tasks that comes from next-of-kin. We have had next-of-kin who have sat by the bed for several days. When we ask them why, they respond that it is because they don’t dare to leave the patient. They have seen the pace we have. (Cancer nurse)

When the nurses were asked what they ask next-of-kin to do, they responded that next-of-kin are not given medical tasks. The nurses seemed reluctant to talk about this:

We can probably not say that we give them (next-of-kin) medical tasks in a way, but they help with safety, care, showers and other such things. Not so much the medical care really, but they might help with giving medications. Pills. (Cancer nurse)

Healthcare professionals described the balance between involving and using next-of-kin as a practical resource as a ‘grey zone’. Nurses asked next-of-kin to perform some tasks because they wanted to involve them, but mostly because the nurses did not have the time or staffing. The results indicate that healthcare professionals depend on next-of-kin in care provision due to understaffing and peak problems.

### Educational challenge

#### Limited systematic next-of-kin education

The interviews with healthcare professionals in Hospital A revealed that there is literally no room for professional updates for managers or healthcare professionals in the nursing group.

Quality champions in the department must work hard to get the management to prioritize academic and research projects. The managers struggled with patient overload and shift coverage with experienced personnel.

The cancer department at Hospital A provided education to breast cancer patients, but they did not invite next-of-kin. No formal education was offered to next-of-kin and next-of-kin was not a topic in the newly designed courses. In Hospital B we found a more stable workgroup of cancer nurses. More attention was paid to learning and education, but as in Hospital A there was no formal education for the next-of-kin. Both hospitals seldom used next-of-kin experiences in courses or education, but they often discussed ethical aspects in patient care and individual needs in the ward on a daily basis. The registrars in Hospital A were pleased with the education activities. They reported that consultants were good role models and accessible, and there was room for professional updates. The registrars in Hospital B were seldom included in difficult patient meeting as part of their professional training, and did not experience increased professional responsibility in parallel with increased professional experience:

You feel that you are stagnating a bit. You have to stay so long on the little less challenging operating level. You dream of more treatment responsibilities and having your own patients. (Registrar)

### The emotional challenge

#### Unspoken expectations of next-of-kin performance and emotional stress

In this category, the next-of-kin role is described as an emotional difficulty from the nurses’ perspective. In both hospitals, next-of-kin were invited to accompany the patient to treatment or information meetings. However, the result showed that the nurses were unclear about the role of next-of-kin. Healthcare professionals expect next-of-kin to be active and participating, but do not articulate this to them:

It is not said out loud, but basically you have expectations once they (next-of-kin) are there. (…) That they try to be active in their role, and not just sit passively by the patient and expect something of us. (Cancer nurse)

In the interviews, there are several examples of next-of-kin sitting at bedside for days due to concerns with medication or staffing. This was difficult for healthcare professionals to resolve. When nurses and consultants receive critical feedback they often took it personally, even if the criticism was directed at the system.

Managers, especially in Hospital A, reported spending a lot of time handling emotional stress among staff. The healthcare professionals experienced emotional stress because they set the priorities, and even if done correctly, it could still feel wrong.

### Physical and technological challenge

#### Location and infrastructure affect possibilities for next-of-kin involvement

Both hospitals had too many patients for their capacity. Healthcare professionals sometimes need to ask next-of-kin to leave the room or to be quiet, because of overcrowded rooms.

In the interviews in Hospital B, informants noted that having too many next-of-kin in small rooms could increase emergency risk and complicate an evacuation. Next-of-kin involvement in general was easier in single room wards. In Hospital B we found two inpatient wards that had different designs. One ward was new and designed with single rooms and two double rooms, with additional bathrooms. A room was reserved for next-of-kin to nap or take breaks. In the other ward, we found rooms designed for four patients with one small bathroom in the hallway. The healthcare professionals claimed that next-of-kin involvement was much easier in the new ward than in the hospital room designed for four patients:

It is too little space. It can affect patient safety. (…). It is too many patients and next-of-kin in one room. There is not enough equipment. You need to use a lot of time to look for equipment and to find a place. We have to take what we find, because there is not enough room for everybody. (Manager)

The results also showed that the documentary system did not include designated areas for documentation of information or correspondence with next-of-kin. Healthcare professionals often spent a lot of time figuring out what information next-of-kin had received, their resources, the patient’s network, and how next-of-kin were involved in the cancer care process.

### Areas of next-of-kin involvement in hospitals

**Fig. 3:**
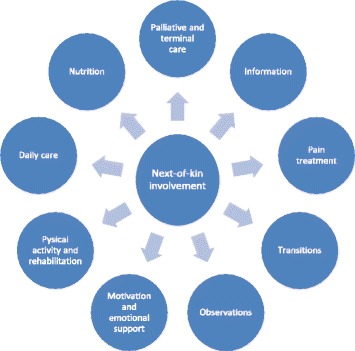
Model 1: Important areas for next-of-kin involvement

Healthcare professionals and managers in this study identified nine areas in which next-of-kin are involved in improving quality and safety (Fig. 3: Model 1). These nine areas were common across the two hospitals, organizational levels and professions within the cancer departments. Next-of-kin were involved in terms of having key information about the patient, for motivating patients during treatment and taking on responsibilities and work tasks related to nutrition, medication, and rehabilitation. Also in transitional care, healthcare professionals depended on involvement of next-of-kin to ensure sound transfer between care levels. During both hospital stay and between care levels next-of-kin were involved in care provision as resources in observation of patients, in parts of daily care, and particularly during palliative and terminal care.

#### Model 1: Important areas for next-of-kin involvement

Some of these areas were described as more natural for next-of-kin to be involved in (information, motivation and palliative/terminal care) considering the close relationship with the patient. But healthcare professionals describe that there is a fine line between being involved for the patients and next-of-kins` best, or being involved because of capacity problems in the department (pain treatment, observation, daily care). Next-of-kin involvement is described as a sensitive area that requires more attention and these nine areas, identified by healthcare professionals and managers, can be used by the hospital cancer care to develop a more thorough understanding of next-of-kin’s role and contribution.

## Discussion

### Similarities and differences between hospitals

This study of managers’ and healthcare professionals’ views and experiences with next-of-kin involvement in cancer care, substantiate a link between next-of-kin involvement, clinical safety, quality improvement, and patient outcome. The role of user involvement to promote better patient outcomes are highlighted by the Institute of Medicine [6] and involvement is also highlighted as a key resource in quality and safety improvement in national government documents [9, 10]. At the meso level, managers at both sites commented on the national government’s change in the next-of-kin policy towards stronger and more involvement. However, there was some confusion over what this involvement should consist of. This was because managers and healthcare professionals claimed that in cancer care there is already a close interaction among the patient, healthcare professionals and next-of-kin. More surprisingly, our study showed that the close interaction with next-of-kin did not follow a structured approach or method of involvement. Consistent with findings in other studies [1, 2], healthcare services and cancer care lack knowledge about methods for involvement. Moreover, this study showed that healthcare professionals at both hospitals lacked knowledge and awareness of next-of-kin role and contribution to quality and patient safety. This calls for a change in national next-of-kin policy. In cancer care, attention should not only be targeted to more or stronger involvement but rather on how healthcare professionals can customize next-of-kin support for each patient. Cancer care departments could also benefit from a more tailored next-of-kin involvement with additional training in e.g. pain management or nutrition (Fig. 3: model 1). This can contribute to increased awareness of the responsibility and work done by the next-of-kin, not only for the patient, but also for the healthcare services.

Findings in this study show that the cancer care provision depends on next-of-kin involvement and collaboration as patients move across service levels. This is not only because of shortage of staff or personnel, but because next-of-kin are sources of valuable knowledge and make a contribution to the patient’s ability to handle and recover from cancer treatment. Next-of-kin cannot be replaced by other stakeholders, and our study parallels other studies showing that next-of-kin takes on tasks for which they are unprepared [19, 20]. Other studies of transitional care equals to ours in terms of the important role of next-of-kin as information carrier [15]. Despite the close interaction between healthcare professionals and next-of-kin we found in the study, next-of-kin were seldom included as an equal part of the care team. Similar results from a Norway have been described by Wiig and colleagues in maternity care [37].

In addition, healthcare professionals and managers in our study emphasized that the next-of-kin carry a heavy burden that can affect their health, work, and family life [5, 38]. Benefits of a more structured approach to next-of-kin involvement in hospital cancer care such as a guide can be twofold. First, it may lighten the next-of-kin’s burden by dividing tasks between healthcare professionals and other stakeholders. Second, it may increase the awareness of next-of-kin’s role in improving the quality and safety of cancer care.

Both hospitals had a positive emotional and cultural environment with strong commitment to patient and next-of-kin. Collective values, interdisciplinary collaboration and commitment acknowledged the role of next-of-kin in the cancer care trajectory. Still, this is not enough to involve next-of-kin appropriately. Despite internally motivated clinical engagement, findings showed that lack of continuity, frustration with interdisciplinary collaboration, external demands and critical feedback from next-of-kin were emotionally stressful for the healthcare professionals. It is important to ask if this emotional stress influences healthcare professionals’ clinical performance in cancer care. Some studies have argued that healthcare professionals’ feelings can compromise patient safety [39, 40], while other studies indicate that oncology nurses’ vigilance can affect patient safety, and the appropriate involvement of next-of-kin may allow nurses to be more vigilant [41] .

The physical and technological challenge stood out as the main significant difference between the two hospitals. In Hospital B we found that workplace conditions (e.g., four-bed rooms and limited space) which both managers and healthcare professionals experienced reducing their abilities to involve next-of-kin in cancer care. These findings are consistent with Bate et al.’s study [25] of the functional physical working environment as a foundation for quality improvement work.

This study has highlighted nine important areas for next-of-kin involvement in hospital cancer care (Fig. 3: model 1). This new knowledge might be helpful for managers and healthcare professionals to develop, explore, and create interventions or methods related to each of the nine areas. Moreover, the nine areas can stimulate a discussion at the macro level about what stronger involvement of next-of-kin should look like. Based on our findings, the discussion should be directed to more structured approaches for next-of-kin involvement. In addition, the next-of-kin policy in Norway does not make a distinction between being next-of-kin in cancer care or other diagnostic fields [9–11]. Our study indicates that there may be a significant difference*.* Next-of-kin involvement in cancer care could be treated as a separate group in terms of developing interventions, methods and guidance for involvement in quality and safety improvement related to the nine areas identified in our study (Fig. 3: Model 1). Even though the results stem from cancer care, the nine areas in Fig. 3: Model 1 might be transferrable to other diagnostic fields, especially in the Nordic countries, due to similarities in organizing of healthcare systems [42].

### Organizing next-of-kin involvement in cancer care – Suggestion for framework development

**Fig. 4:**
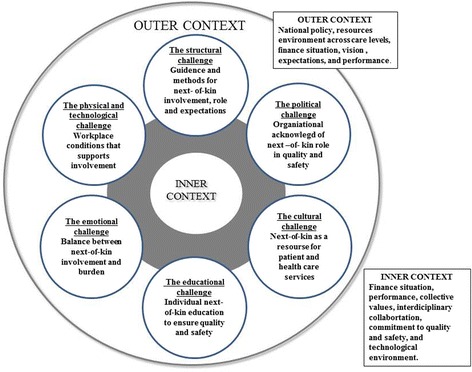
Model 2: Revised framework model inspired by [25]

Our findings bring a new perspective to next-of-kin’s role in and contribution to the cancer care trajectory. They demonstrate the complexity of hospital organizational context and how it affects healthcare professionals and managers in their daily meetings with next-of-kin. The Organizing for Quality framework was applied when analyzing the qualitative data according to the six common challenges [25]. Anchored in our findings, we suggest further development of the framework shown in Fig. 4: Model 2. We specify areas of key importance for next-of-kin involvement under each challenge to elaborate and specify content to the six challenges. By doing this, we simplify application of the framework in a stakeholder perspective in research and everyday clinical practice [26, 30]. Our development is relevant for future predictions and prospects for governments, the research field, managers and healthcare professionals to strengthen the dimension of next-of-kin involvement in improvement of hospital cancer care. The six challenges were common across the two case hospitals, but should be tested by managers and healthcare professionals in a larger sample of hospital cancer care settings in Norway and an international context [25, 32].

#### Model 2: Revised framework model inspired by [25]

We suggest that the structural challenge is to build competence on what a more structured approach (guidance and methods) can contribute with for making the cancer care journey better and safer for the patient. The political- and cultural challenge needs to include organizational acknowledgement of the role next-of-kin holds in quality and safety work and acknowledge next-of-kin as a potential resource for both the patient and healthcare services. The educational challenge, needs to create educational activities to support the next-of-kin role, resources, and ability to master and adapt to the cancer journey. The emotional challenge is to strike a balance between next-of-kin involvement and next-of-kin burden. Our study shows that no cancer journey is free of burdens, but there could be a mutual obligation to ensure that the division of work is balanced among all stakeholders, including next-of-kin. With regards to the physical and technological challenge, there is a need to ensure that locations and workplace conditions support next-of-kin involvement.

### Limitations

The two hospitals were selected because they have the same external context, are similar in structure, location and belong to the same RHA. Based on the sample, we cannot illuminate variations, for instance if we selected hospitals based on good or poor performance, or not within the same RHA. We explored only two hospitals. A larger sample could have generated different findings. This study did not include next-of-kin. Their perspective is covered in another stage of the project. This articles` main focus was a manager and healthcare professional viewpoint. However, with the limitations in mind, we have described and derived meaningful insight and a new perspective on the next-of-kin role in quality and safety improvement with a multilevel approach (macro, meso, micro). We are confident that this approach will contribute to understanding the next-of-kin’s role in improving quality and safety in cancer care.

## Conclusion

In this study, we have explored the influence of next-of-kin involvement in quality and safety improvement within cancer care in two hospitals. The study shows that next-of-kin holds an important safety dimension in patient-centred care [6] and demonstrates a close interaction and collaboration among patient, next-of-kin, and healthcare professionals in cancer care. However, there were no systematic approaches, strategies and plans for next-of-kin involvement. The perceived challenges that healthcare professionals described were closely connected to hospital context, workplace conditions and awareness of next-of-kin involvement as a resource for quality and safety improvement. Based on descriptions across the two case hospitals, care levels, and professions, we identified nine areas (Fig. 3: Model 1), where next-of-kin are important stakeholders in improving quality and safety (nutrition, palliative and terminal care, information, pain treatment, transitions, observations, motivation and emotional support, physical activity and rehabilitation, and daily care). Next-of-kin were silent external partners in the medical team around the patient that often had significant responsibilities. Their knowledge was used by the healthcare professionals, but they were seldom acknowledged in the same way as the other stakeholders around the patient with regards to education, guidance, or other systematic means of involvement.

Future research steps and clinical implication for next-of-kin involvement could benefit from using the suggested revision of the Organizing for Quality framework (Fig. 4: Model 2) to develop organizational procedures or as a basis for evaluating how different healthcare organizations practice next-of-kin involvement. Additional studies should include next-of-kin experiences and perspectives on how they would like to be involved in improving quality and safety in cancer care. Finally, future research should investigate how a more structured approach to next-of-kin involvement in cancer care, such as a guide or checklist, influence patient outcome and reduction in next-of-kin burden.

## Abbreviation

RHA: Regional Health Authority
